# Information Infrastructure for Cooperative Research in Neuroscience

**DOI:** 10.1155/2009/409624

**Published:** 2009-06-01

**Authors:** P. J. Durka, G. J. Blinowski, H. Klekowicz, U. Malinowska, R. Kuś, K. J. Blinowska

**Affiliations:** ^1^Department of Biomedical Physics, Warsaw University, Hoza 69 street, 00 681 Warszawa, Poland; ^2^Institute of Computer Science, Warsaw University of Technology, Nowowiejska 15/19 street, 00 665 Warszawa, Poland

## Abstract

The paper describes a framework for efficient sharing of knowledge between research groups, which have been working for several years without flaws. The obstacles in cooperation are connected primarily with the lack of platforms for effective exchange of experimental data, models, and algorithms. The solution to these problems is proposed by construction of the platform (EEG.pl) with the semantic aware search scheme between portals. The above approach implanted in the international cooperative projects like NEUROMATH may bring the significant progress in designing efficient methods for neuroscience research.

## 1. Introduction

Nowadays, publications alone are not enough to coherently increase our knowledge of the mathematical methods applied in neuroscience. To foster the progress on that field, the efficient mechanisms of sharing the experience of scientific teams are needed. The NEUROMATH is an action in which the scientists are called to harmonize their efforts in order to offer a comprehensive approach to the problem of the estimation of brain activity and connectivity for sensory and cognitive behavioral tasks. For solving this problem, the optimal mathematical methods has to be designed and tested on the large databases, which require efficient mechanisms for sharing resources. The problem of an efficient application of internet databases for sharing computational resources was approached, for example, in [1] where the practical barriers to progress on that field were identified. 

This paper proposes working solutions to these issues, implemented and working for several years in the EEG.pl portal with the semantic-aware search scheme for interconnecting portals. The structure and layout of EEG.pl (except for the interportal search), at least of the part dedicated to sharing software, can be found in the recently started Software Center of the International Neuroinformatics Coordination Facility (http://software.incf.org/). When adopted within the NEUROMATH framework, these solutions will foster the cooperation between the groups and consolidate their efforts to the aim of designing the optimal methods for estimation of brain activity and connectivity.

## 2. EEG.pl Open Repository

EEG.pl is a portal dedicated to sharing software, models, and data related to EEG and local field potentials. It is open to anybody interested in making relevant items freely available or downloading resources shared by others. Only submission of material requires free registration; browsing and downloading is available to anybody. The invitation on the first page states:

*EEG.pl is an open repository for software, publications and datasets related to the analysis of brain potentials: electroencephalogram (EEG), local field potentials (LFPs) and event related potentials (ERP), created to foster and facilitate Reproducible Research in these fields.*

*You can freely search the content of this and other thematic vortals linked via the Interneuro initiative. As a registered user you can submit your article, data or model. Registration and submissions are free. You can also comment and respond to comments on any of the published items.*

There are also Disclaimers: *none of the organizations or individuals supporting or maintaining this site is responsible for the content provided by users and any damage which may result from its application. In particular, we do not provide any virus scanning for the binaries available as “software”. We do not peer-review submitted material, just retain the right to reject irrelevant or low quality submissions. We believe in opinions of the Neuroscience Community, expressed hereby in the comments which users can attach to any of the published items. We believe that these comments provide most objective evaluation*.

During over five years of experience in running this service, we learned two major lessons.

1. The software framework and chosen solutions are stable and caused no problems while retaining large amount of flexibility to both the administrator and the users.
2. EEG.pl is not the only resource of this kind, and the response of the community was not as widespread as expected.

The latter issue calls into attention the issue of interoperability with other portals. This can be achieved within the “Interneuro” framework, described in http://www.eeg.pl/documents/about_connections. Below we briefly recall the ideas underlying the semantic-aware search, which is the key feature in this scheme.

## 3. Semantic Aware Search

Semantic-aware search—contrary to the search provided by typical Internet-wide search engines like Google—indexes not only simple keyword data but also the *meaning* of the data. In case of books, that metainformation would include the author, creator, title, major keywords, and references. In general, the choice of metainformation is not trivial. Fortunately standards exist which regulate naming and scope of metainformation attributes. One of the most popular standards in this field is the Dublin Core (DC) standard. The DC specification is developed and maintained by “The Dublin Core Metadata Initiative” (DCMI), an “open forum engaged in the development of interoperable online metadata standards that support a broad range of purposes and business models.” The full specification of the DC standard may be found in [2]. Here we will summarize only the most important elements of the DC metadata.

Type * *“The nature or genre of the content of the resource”—this may be a text (paper, article, preprint); a software item (i.e., a description of a freeware or commercial software piece); a dataset (i.e., an experiment collected time series in a well know format).
Title * *“A name given to the resource”, for example, in case of a paper—its title.
Identifier * *“An unambiguous reference to the resource within a given context”; the identifier does not have to have a sensible meaning to a human being; “it is simply a unique token identifying the resource”, for example, a URL.
Creator * *“An entity primarily responsible for making the content of the resource”—that is, a person, an organization, or a service.
Description * *“An account of the content of the resource”—abstract, table of contents, reference, and so forth.
Subject * *“The topic of the content of the resource” —keywords, key phrases, and classification codes that describe the resource.

DC defines also a handful of other attributes, like time and date information, information about the publisher, more data about the content itself, and so forth. Sophisticated distributed search mechanism is around it. With metainformation standardized, there is no longer an issue of“what to search for?”, only an issue of “how to search?” (technically) remains. 

For the low-level implementation of queries we have adopted the SOAP/RDF XML [3] based standards for describing queries, and results. As a consequence, the HTTP protocol [4] is used for transporting the query and the response over the network. 

The search service is build around the distributed P2P paradigm: each portal is both a client and a server, that is, is able to formulate and send the queries as well as listen for search requests and to answer them. The rationale for using SOAP/RDF/XML is the following.

- SOAP/XML is portable and both platform, and system independent.
- SOAP/XML and SOAP over http are *defacto* standards for building distributed applications.
- SOAP is simple—there is no heavyweight software required to generate and parse it.
- There is a multitude of XML parsers and tools available (both commercial and open-source), so building software compatible with our format should not be a technical problem.

**Figure 1:**
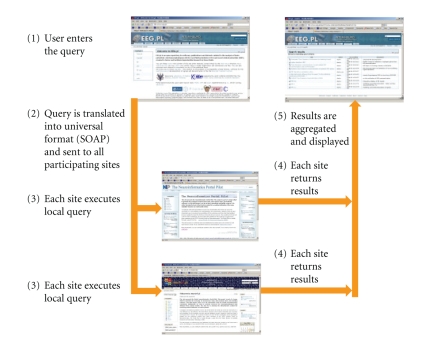
Information flow during the distributed search according to the InterNeuro scheme.

The process of executing a distributed query, illustrated also on Figure 1, is executed as follows.

1. User enters the query: he/she connects to one of the cooperating sites (e.g., http://eeg.pl), chooses “advanced search”, enters the search phrase(s), marks the “external search” check box, and clicks the search button.
2. Query is translated into universal format (SOAP/XML) and sent to all participating sites.
3. Each site executes local query.
4. Each site returns results.
5. Results are aggregated and displayed to the user.

**Figure 2:**
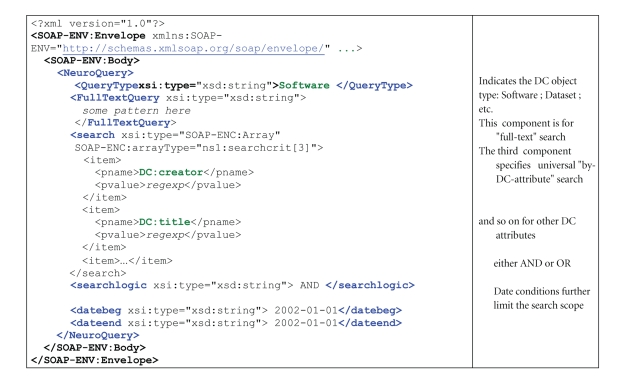
SOAP query.

The format of queries and returned results is based upon (Simple Object Access Protocol SOAP) [3]—a stateless, message exchange paradigm based on XML. In simpler terms, SOAP is a mechanism similar to (Remote Procedure Call RPC) based on open standards: the remote object access (or a “procedure call”) is express purely in XML notation; the same applies to returned results. A SOAP message consists of an outermost envelope, an optional header, and body. From the logical point of view the body consists of a remote objects' (or procedures') identifier and parameters. The SOAP standard describes how parameters should be represented, serialized, and encoded. SOAP defines both a method for encoding simple types (strings, integers, etc.) as well as complex types such as arrays and structures. In case of the remote search employed in Interneuro a relatively simple query is used: only string type parameters representing DC attributes are passed—see Figure 2. 

**Figure 3:**
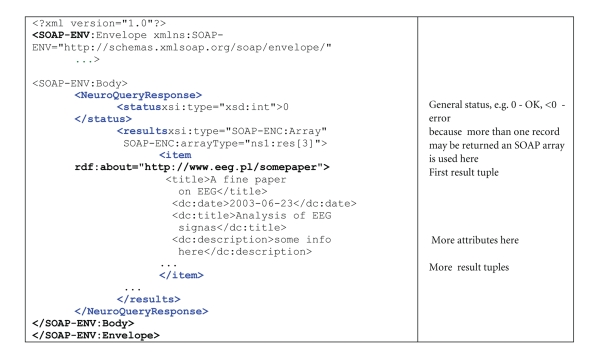
RDF response to the query from Figure 2.

The result is generated and recorded as an RDF serialized (encoded) in SOAP response—see Figure 3. RDF stands for Resource Description Framework [3], a language for representing information about resources in the World Wide Web. RDF, similarly to SOAP, is based on XML. It is particularly intended for representing metadata about web resources, such as the title, author, and modification date of a web page. RDF is intended for situations in which information needs to be processed by applications, rather than being only displayed for people. RDF provides a common framework for expressing this information so it can be exchanged between applications without loss of meaning.

Implementation of the EEG.pl portal is based on the Zope/CMS/Plone (http://plone.org/) free application server/content management/portal engine. Although Zope/Plone provides some mechanisms for distributed communication between different sites (RPC-over-XML) it currently lacks SOAP/RDF support as such. We have used ZOPE's template mechanisms and programming capabilities to develop a distributed search component. The software is written in Python (a default development language for ZOPE, in which the whole system is actually written) and freely available as ZOPE package (technically ZOPE “product”). These software components are freely available from http://eeg.pl.

## 4. Conclusion

We presented a working solution to some of the problems encountered in the integration of the efforts of scientific teams, such as the participants of the NEUROMATH action. Proposed approach answers the need of a computational platform for sharing resources.

EEG.pl portal and the semantic-aware search scheme provide a solution to the major problem of information noise, which sometimes overweight advantages of the Internet in scientific communication. Our solution lies in between the two extrema of the absolute centralization and a complete decentralization. Disadvantages of one central repository of information are obvious, but, on the other hand, Semantic Web and superintelligent software agents, creating structure from the chaos, are still more of buzzwords than reality. We propose a humble compromise. As presented, relevant information can be gathered in specialized repositories of possibly well-defined scope. Owing to this specialization, these relatively small services can assure the quality and proper annotation of resources. Seamless integration of these small repositories into a significant knowledge base can be effectuated by the connection paradigm presented in this paper. More technical details and a complete software implementation of this solution are freely available from http://eeg.pl.

## References

[1] Blinowska KJ, Durka PJ (2005). Efficient application of internet databases for new signal processing methods. *Clinical EEG and Neuroscience*.

[2] Powell A, Nilsson M, Naeve A, Johnston P, Baker T DCMI Abstract Model. http://dublincore.org/documents/abstract-model.

[3] Gudgin M, Hadley M, Mendelsohn N SOAP Version 1.2 Part 1: Messaging Framework, W3C Recommendation. http://www.w3.org/TR/soap12-part1.

[4] Fielding R, Gettys J, Mogul  J Hypertext Transfer Protocol—HTTP/1.1. http://www.w3.org/Protocols/rfc2616/rfc2616.html.

